# A Predictive Model and Scoring System for All-Cause Mortality in Patients With Atrial Fibrillation Combined With Obstructive Sleep Apnea Syndrome: A Retrospective Case–Control Study

**DOI:** 10.31083/RCM36467

**Published:** 2025-06-25

**Authors:** Xiaoting Zhang, Meng Wei, Pengjie Xue, Yanmei Lu, Baopeng Tang

**Affiliations:** ^1^Department of Cardiac Pacing and Electrophysiology, The First Affiliated Hospital of Xinjiang Medical University, 830000 Urumqi, Xinjiang, China; ^2^Xinjiang Key Laboratory of Cardiac Electrophysiology and Cardiac Remodeling, The First Affiliated Hospital of Xinjiang Medical University, 830000 Urumqi, Xinjiang, China

**Keywords:** atrial fibrillation, catheter ablation, hypoxemia, obstructive sleep apnea syndrome

## Abstract

**Background:**

The high prevalence and mortality rate of combined atrial fibrillation (AF) and obstructive sleep apnea syndrome (OSAS) impose a significant disease burden on public healthcare systems. However, there is currently a lack of risk-assessment tools for all-cause mortality in patients with both AF and OSAS. Therefore, this study utilized clinical data from patients at the First Affiliated Hospital of Xinjiang Medical University to establish a predictive model and address this gap.

**Methods:**

This study included 408 patients with AF and OSAS, randomly divided into a training set (n = 285) and a validation set (n = 123). Subsequently, the training set was split into deceased and surviving groups to analyze in-hospital indicators.

**Results:**

A total 10 variables were selected from an initial 64 variables in patients with AF and OSAS identified through Lasso regression screening, including hypoxemia, catheter ablation (CA), red blood cell count (RBC), lymphocyte count, basophil granulocyte count, total bile acids, D-dimer, free triiodothyronine, N-terminal pro-brain natriuretic peptide (NT-proBNP), and chronic obstructive pulmonary disease. Variables identified as significant in the univariate logistic regression analysis were included in the multivariable logistic regression analysis, which revealed that CA (odds ratio (OR) = 0.21) was an independent protective factor. In contrast, moderate-to-severe hypoxemia (OR = 11.11), RBC <3.8 × 10^12^/L (OR = 20.70), and D-dimer ≥280 ng/mL (OR = 7.07) were independent risk factors. Based on this, receiver operating characteristic (ROC) curves were plotted, showing area under the curve (AUC) values of 0.96 for the training set and 0.91 for the validation set, indicating the model exhibited good predictive ability. A risk-scoring system was developed to assess the overall mortality risk of patients with AF and OSAS. The percentage bar chart demonstrated an increase in mortality rate and a decrease in survival rate as the risk level increased.

**Conclusions:**

The predictive model and risk scoring system developed in this study exhibit good predictive abilities in evaluating all-cause mortality in patients with AF and OSAS, providing valuable clinical guidance and reference.

## 1. Introduction

Atrial fibrillation (AF) is one of the most common cardiac arrhythmias in adults, affecting 2–4% of the global adult population and 1.6% of Chinese adults [1, 2, 3]. Due to its frequent association with advanced age and comorbid chronic conditions (such as hypertension, diabetes, and obesity), the prevalence of AF has been progressively increasing annually [4, 5]. Obstructive sleep apnea syndrome (OSAS) is also a common chronic condition, being the most prevalent sleep-related breathing disorder; it is characterized by recurrent episodes of sleep apnea leading to periodic hypoxemia and hypercapnia [6]. Globally, OSAS affects over a billion people, with some countries experiencing prevalence rates exceeding 50% [7]. AF and OSAS often share common risk factors, including hypertension, coronary artery disease, and congestive heart failure (HF) [8]. The two are closely related, with an AF prevalence of approximately 21–87% among OSAS patients [5], and OSAS being present in about 50% of AF patients [9]. Having both AF and OSAS (AF + OSAS) can worsen patients’ outcomes; with a link between AF and higher mortality rates [10], severe OSAS is significantly associated with an increased risk of all-cause mortality in patients [11]. In addition to the above, AF + OSAS imposes significant economic burdens on patients’ families and the public healthcare system. In the United States, the annual cost of treating AF and its complications exceeds $28 billion [12], and a study in Italy indicated an annual treatment cost of over €234M for patients with OSAS [13].

For many years, antiarrhythmic drugs have been the mainstay of treatment for AF. Catheter ablation (CA), since the introduction of in 1994, has provided AF patients with additional treatment options and hope. A meta-analysis has shown that CA significantly reduces cardiovascular and all-cause mortality rates in AF patients [14], but the recurrence rate after CA in patients with AF + OSAS can be as high as 80% [15]. Continuous positive airway pressure (CPAP) therapy, the primary treatment for OSAS patients, has not yet shown an impact on survival rates in OSAS patients [16]. The effectiveness of current mainstream treatment options in improving all-cause mortality rates in patients with AF + OSAS remains unclear.

As mentioned earlier, both AF and OSAS are significantly associated with increased all-cause mortality rates, with intricate and complex underlying mechanisms. Despite this, effective preventive strategies for all-cause mortality in patients with AF + OSAS have not been established so far, which has raised significant concerns. With the aging population trend increasing the incidence of AF + OSAS, this undoubtedly places a heavy burden on the public healthcare system in terms of disease and economic pressure. To our knowledge, there is currently no dedicated risk-assessment tool for all-cause mortality in patients with AF + OSAS.

As the largest healthcare institution in the region, we have access to the highest quality healthcare resources and the largest patient population. Our medical center serves patients of various races and is equipped with a comprehensive Hospital Information System (HIS). Therefore, our research focused on using routine clinical characteristics, laboratory parameters, and imaging findings readily available in the HIS to construct predictive models and scoring systems. These tools were used to assess the all-cause mortality risk in patients with AF + OSAS, with the goal of optimizing patient prognosis and quality of life. Additionally, by identifying high-risk patients, healthcare professionals can adopt more proactive monitoring and intervention measures. Understanding the mechanisms of all-cause mortality in patients with AF + OSAS and developing new approaches to enhance survival rates is a promising area for future in-depth research.

## 2. Materials and Methods

### 2.1 Study Design and Participants

The present study was a retrospective case-control study that included a total of 446 patients with AF + OSAS who were hospitalized and diagnosed at the First Affiliated Hospital of Xinjiang Medical University from January 1, 2012, to August 31, 2024. The inclusion criteria for the study were: (1) age ≥18 years old; (2) patients with a confirmed diagnosis of AF combined with OSAS. Exclusion criteria were: (1) valvular AF; (2) loss to follow-up data; (3) more than 10% data missing. In the present study, we conducted follow-up contact with the enrolled patients from October 1st to October 7th, 2024. The follow-up procedures were the same for all participants. Among the participants, 25 either changed their contact information or declined follow-up, resulting in a lost follow-up rate of 5.6%. Those individuals were excluded from the analysis. Based on the inclusion and exclusion criteria, the selected patients were randomly allocated to a training set (*n* = 285) or a testing set (*n* = 123). The allocations were designed to maintain a 7:3 ratio.

**Fig. 1.**
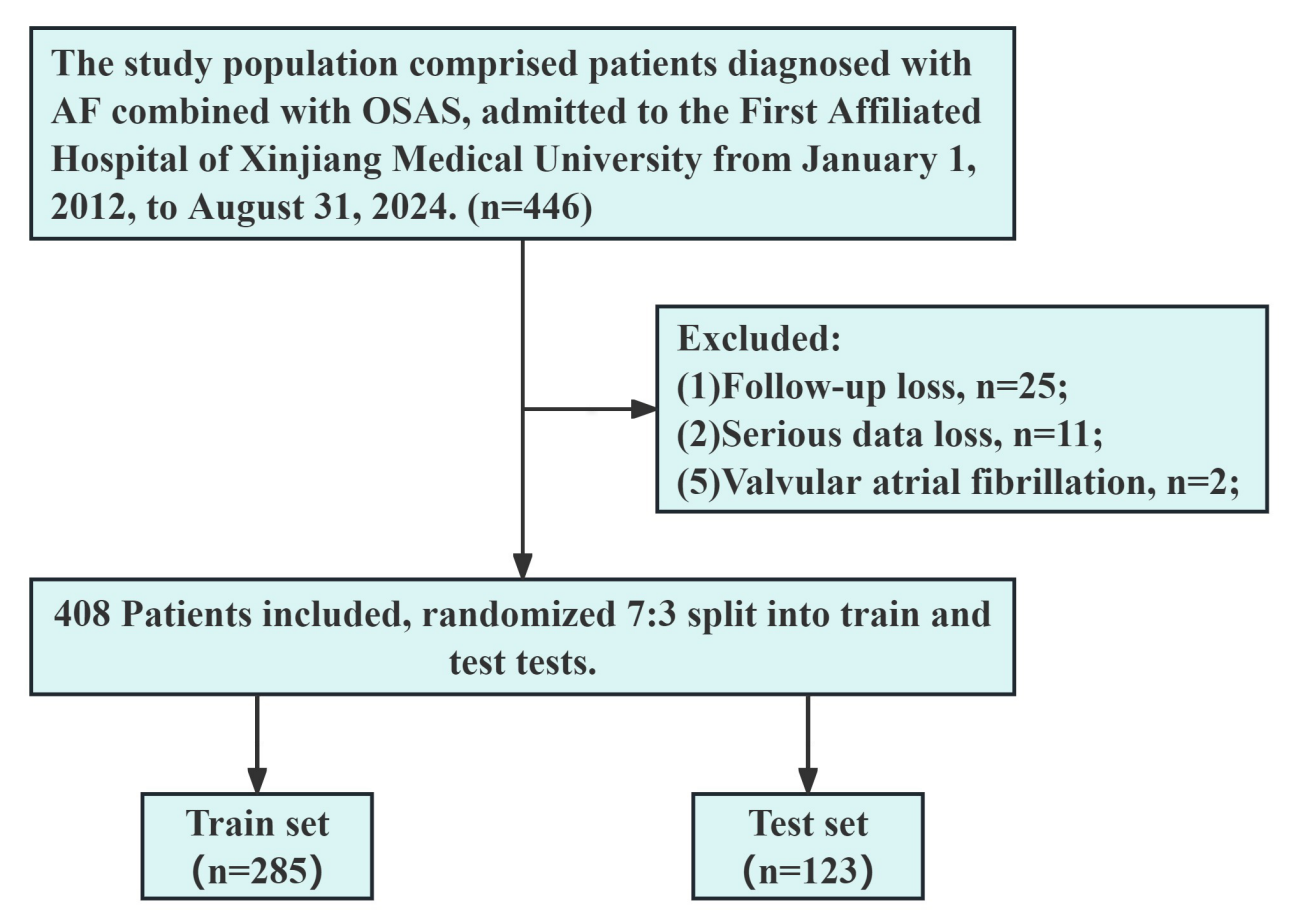
**Flowchart outlining the screening process for study subjects**. OSAS, obstructive sleep apnea syndrome; AF, atrial fibrillation.

This study was approved by the Ethics Committee of the First Affiliated Hospital of Xinjiang Medical University with the ethics review number K202409-07. Due to the retrospective nature of this study and data collection solely through the HIS, the application for exempting informed consent signing was granted by the Ethics Committee of the First Affiliated Hospital of Xinjiang Medical University. Furthermore, patient consent was obtained during telephone follow-ups, and those who did not consent were excluded from the study. The detailed patient selection process is shown in Fig. 1.

### 2.2 Data Collection

The data were collected from the initial hospital admission records of patients with AF + OSAS, including: (1) Basic characteristics: age, sex, smoking, drinking; (2) Underlying diseases: hypertension, coronary artery disease, diabetes, stroke, chronic obstructive pulmonary disease (COPD); (3) Physical examination results: heart rate, lower extremity edema, body mass index, pulse pressure difference ; (4) Examination findings: blood laboratory tests, noting red blood cell count (RBC) and white blood cell counts, coefficient of variation of red blood cell distribution width, counting of lymphocytes, monocytes, neutrophils, eosinophils, basophil granulocyte (Baso), and platelets, hemoglobin; Liver function tests: alanine and aspartate aminotransferases, γ-glutamyltransferase, direct and indirect bilirubin, albumin, globulin, total bile acids (TBA), alkaline phosphatase; Renal function markers such as creatinine, urea, uric acid, cystatin C, glomerular filtration rate; Lipid profile included total cholesterol, triglycerides, high and low density lipoprotein cholesterol; Electrolytes such as potassium, sodium, chloride, calcium, phosphorus, magnesium, glucose; Coagulation panels such as thrombin time, prothrombin time, D-dimer, fibrinogen; Cardiac function indicators comprised N-terminal pro-brain natriuretic peptide (NT-proBNP), creatine kinase, left ventricular ejection fraction, left atrial diameter, right atrial diameter, right ventricle; The function of the thyroid gland contained thyroid stimulating hormone, free triiodothyronine (FT3), free tetraiodothyronine; (5) Other variables of concern included the severity of OSAS and hypoxemia as reflected by polysomnography, and whether they underwent CA.

### 2.3 Study Outcome Events

The outcome of the present study was the all-cause mortality rate of patients with AF + OSAS, which refers to the rate or occurrence of all deaths caused by any reasons during the study period [3]. The follow-up regarding patient mortality status was conducted by telephone contact and HIS.

### 2.4 Statistical Analysis

Statistical analyses were performed using SPSS 22.0 (IBM Corp., Armonk, NY, USA) and R (version 4.2.1; R Foundation for Statistical Computing, Vienna, Austria). R packages (https://cran.r-project.org/) that were used included: glmnet [4.1.7]; pROC [1.18.0]; ggplot2 [3.3.6]; rms [6.4.0]; Resource Selection [0.3–5]; and rmda [1.6]. Statistical analysis of continuous variables was conducted using SPSS, with mean (SD) reported for normally distributed variables and independent sample *t*-tests for variables meeting the conditions for *t*-tests. Skewed variables were described using the median and interquartile range [P50 (P25, P75)], and between-group comparisons were performed using the Wilcoxon-Mann-Whitney test. Categorical variables were described as *n* (%) and between-group comparisons were performed using the Chi-square test or Fisher’s exact probability test.

In the present study, data on a total of 64 variables were collected from patient records. Lasso regression, incorporating an L1 norm constraint into the cost function of the linear regression model, was used. Tuning of the lambda parameter was carried out to aid in variable selection and to adjust complexity. To address high-dimensional data and feature selection for constructing the logistic regression model, 10-fold cross-validation was used. Variable selection was guided by the optimal evaluation index represented by the lambda value (lambda.min). In the Lasso regression, we used a 10-fold cross-validation. We randomly allocated patients in a 7:3 ratio using a random number table, with the random number being 1. We developed the research model using the training set and then validated the model with the testing set. Further details on the diagnostic Lasso coefficient selection process and variable trajectory can be found in the initial section of the results.

The variables that were selected through Lasso regression were incorporated into the study, with dummy variables assigned to the original variables. After that, a univariate logistic regression analysis was performed on the dummy variables to assess the patients’ all-cause mortality rates. Variables exhibiting a *p ≤* 0.05 were subjected to further analysis using multivariate logistic regression. Based on these analyses, a logistic regression model was established, and the receiver operating characteristic (ROC) curve was constructed to evaluate the predictive performance, utilizing the area under the curve (AUC) as a metric.

In this study, decision curve analysis (DCA) was used to assess the predictive performance of the clinical prediction model. Calibration analysis and visualization were performed by constructing a generalized linear model and establishing a binary logistic model to uncover discrepancies between predicted probabilities and actual observed outcomes, thereby evaluating the model’s goodness of fit. A diagnostic nomogram was developed by integrating predictive indicators with scaled line segments, which were plotted on a plane to elucidate the interrelationships among variables within the predictive model. The model’s calibration was evaluated using the Hosmer-Lemeshow Goodness-of-Fit Test.

Using the results of multivariate regression, a risk-score chart for all-cause mortality in patients with AF + OSAS was constructed based on the risk-score functions derived from The Framingham Study [17]. Furthermore, an analysis of score impact was carried out. The α level for statistical significance of *p* ≤ 0.05 was established for all methods used in this study.

## 3. Results

### 3.1 Using Lasso Regression for Preliminary Data Cleaning

**Fig. 2.**
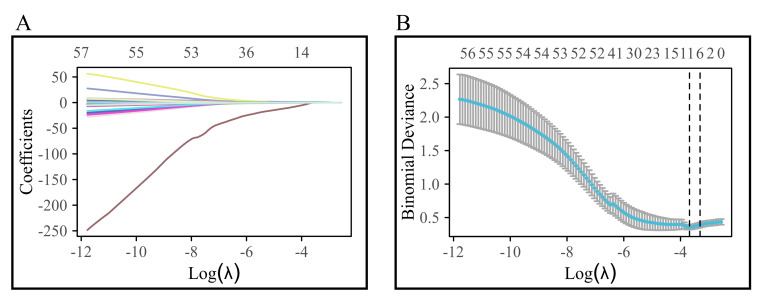
**Variable trajectory and selection process of Lasso regression**. (A) shows the variable trajectory diagram; (B) shows the selection flowchart of Lasso regression.

After Lasso regression selection, 10 variables were chosen from the initial 64 variables of patients with AF + OSAS, comprising: Hypoxemia, CA, RBC count, lymphocyte count, Baso count, TBA, D-dimer, FT3, NT-proBNP, and COPD. The variable trajectory and selection process of Lasso regression are illustrated in Fig. 2.

### 3.2 Comparison of Training and Testing Sets in Patients With AF + OSAS

Comparisons revealed no differences in all-cause mortality, CA, RBC count, lymphocyte count, Baso count, TBA, D-dimer, FT3, NT-proBNP, or COPD, both before and after assignment, between the training and testing sets. There were 23 patient deaths and 385 patient survivals in this study. The mortality rate of patients with AF and OSAS was 5.64%, with all-cause mortality rates of 6.67% in the training set and 3.25% in the testing set. Details of the assignment can be found in Table 1, and Tables 2,3 provide a comprehensive comparison between the training and testing sets.

**Table 1.** **Dummy variable assignment table for each variable**.

Variables	Rules for assigning dummy variables
All-cause mortality	No = 0, Yes = 1
Hypoxemia	Mild = 0, Moderate & Severe = 1
CA	No = 0, Yes = 1
RBC, 10^12^/L	[3.8, 5.1] = 0, <3.8 = 1, >5.1 = 2
Lymphocyte, 10^9^/L	[1.1, 3.2] = 0, <1.1 = 1, >3.2 = 2
Baso, 10^9^/L	≤0.06 = 0, >0.06 = 1
TBA, µmol/L	≤10 = 0, >10 = 1
D-dimer, ng/mL	<280 = 0, ≥280 = 1
FT3, pmol/L	[3.1, 6.8] = 0, <3.1 = 1, >6.8 = 2
NT-proBNP, ng/L	≤125 = 0, >125 = 1
COPD	No = 0, Yes = 1

Notes: CA, catheter ablation; RBC, red blood cell; Baso, basophil granulocyte; TBA, total bile acids; FT3, free triiodothyronine; COPD, chronic obstructive pulmonary disease; NT-proBNP, N-terminal pro-brain natriuretic peptide.

**Table 2.** **Comparison of original data between training and testing sets of patients with AF + OSAS**.

Variables		Total (n = 408)	Test (n = 123)	Train (n = 285)	*t*/Z/χ^2^	*p*
RBC, mean (SD)		4.76 (0.62)	4.80 (0.63)	4.74 (0.61)	0.81	0.42
Lymphocyte, Med (Q_1_, Q_3_)		1.91 (1.49, 2.32)	1.91 (1.52, 2.38)	1.91 (1.48, 2.28)	–0.67	0.504
Baso, Med (Q_1_, Q_3_)		0.03 (0.02, 0.05)	0.03 (0.02, 0.04)	0.03 (0.02, 0.05)	–0.34	0.734
TBA, Med (Q_1_, Q_3_)		3.89 (2.21, 6.38)	3.58 (2.08, 6.34)	3.89 (2.29, 6.42)	–0.65	0.518
D-dimer, Med (Q_1_, Q_3_)		84.00 (48.00, 198.50)	84.00 (51.00, 207.00)	84.00 (46.00, 181.00)	–0.94	0.348
FT3, Med (Q_1_, Q_3_)		4.57 (4.08, 5.11)	4.57 (4.05, 5.11)	4.57 (4.10, 5.11)	–0.25	0.803
NT-proBNP, Med (Q_1_, Q_3_)		798.00 (181.25, 1945.75)	847.00 (195.00, 1896.00)	737.00 (182.00, 1960.00)	0.00	1
Death, *n* (%)					1.88	0.17
	No	385 (94.36)	119 (96.75)	266 (93.33)		
	Yes	23 (5.64)	4 (3.25)	19 (6.67)		
Hypoxemia, *n* (%)					0.17	0.682
	Mild	163 (39.95)	51 (41.46)	112 (39.30)		
	Moderate & Severe	245 (60.05)	72 (58.54)	173 (60.70)		
CA, *n* (%)					0.02	0.894
	No	144 (35.29)	44 (35.77)	100 (35.09)		
	Yes	264 (64.71)	79 (64.23)	185 (64.91)		
COPD, *n* (%)					0.28	0.598
	No	379 (92.89)	113 (91.87)	266 (93.33)		
	Yes	29 (7.11)	10 (8.13)	19 (6.67)		

Notes: SD, standard deviation; Med, median; Q_1_: 1st Quartile; Q_3_: 3st Quartile; *t*: *t*-test; Z: Mann-Whitney test; χ^2^: Chi-square test; CA, catheter ablation; RBC, red blood cell; Baso, basophil granulocyte; TBA, total bile acids; FT3, free triiodothyronine; COPD, chronic obstructive pulmonary disease; AF, atrial fibrillation; OSAS, obstructive sleep apnea syndrome.

**Table 3.** **Comparison of dummy variable assignment between training and testing sets of patients with AF + OSAS**.

Variables		Total (n = 408)	Test (n = 123)	Train (n = 285)	χ^2^	*p*
CA, *n* (%)					0.02	0.894
	No	144 (35.29)	44 (35.77)	100 (35.09)		
	Yes	264 (64.71)	79 (64.23)	185 (64.91)		
Death, *n* (%)					1.88	0.17
	No	385 (94.36)	119 (96.75)	266 (93.33)		
	Yes	23 (5.64)	4 (3.25)	19 (6.67)		
Hypoxemia, *n* (%)					0.17	0.682
	Mild	163 (39.95)	51 (41.46)	112 (39.30)		
	Moderate & Severe	245 (60.05)	72 (58.54)	173 (60.70)		
RBC, *n* (%)					0.71	0.702
	[3.8, 5.1]	278 (68.14)	87 (70.73)	191 (67.02)		
	<3.8	17 (4.17)	4 (3.25)	13 (4.56)		
	>5.1	113 (27.70)	32 (26.02)	81 (28.42)		
Lymphocyte, *n* (%)					0.72	0.699
	[1.1, 3.2]	362 (88.73)	111 (90.24)	251 (88.07)		
	<1.1	30 (7.35)	7 (5.69)	23 (8.07)		
	>3.2	16 (3.92)	5 (4.07)	11 (3.86)		
Baso, *n* (%)					0.11	0.746
	≤0.06	372 (91.18)	113 (91.87)	259 (90.88)		
	>0.06	36 (8.82)	10 (8.13)	26 (9.12)		
TBA, *n* (%)					0.77	0.38
	≤10	374 (91.67)	115 (93.50)	259 (90.88)		
	>10	34 (8.33)	8 (6.50)	26 (9.12)		
D-dimer, *n* (%)					0.01	0.94
	<280	326 (79.90)	98 (79.67)	228 (80.00)		
	≥280	82 (20.10)	25 (20.33)	57 (20.00)		
FT3, *n* (%)					-	0.656
	[3.1, 6.8]	382 (93.63)	114 (92.68)	268 (94.04)		
	<3.1	25 (6.13)	9 (7.32)	16 (5.61)		
	>6.8	1 (0.25)	0 (0.00)	1 (0.35)		
NT-proBNP, *n* (%)					0.03	0.865
	≤125	75 (18.38)	22 (17.89)	53 (18.60)		
	>125	333 (81.62)	101 (82.11)	232 (81.40)		
COPD, *n* (%)					0.28	0.598
	No	379 (92.89)	113 (91.87)	266 (93.33)		
	Yes	29 (7.11)	10 (8.13)	19 (6.67)		

Notes: χ^2^, Chi-square test; -, Fisher exact; CA, catheter ablation; RBC, red blood cell; Baso, basophil granulocyte; TBA, total bile acids; FT3, free triiodothyronine; COPD, chronic obstructive pulmonary disease; AF, atrial fibrillation; OSAS, obstructive sleep apnea syndrome; NT-proBNP, N-terminal pro-brain natriuretic peptide.

### 3.3 Comparison of Variable Characteristics Between Survival and Death Groups of Patients With AF + OSAS in the Training Set

In the training set, there were 19 patients in the dead group and 266 patients in the surviving group. Comparison of the original variables between the two groups showed that the dead group had higher levels of D-dimer, NT-proBNP, the proportion of moderate to severe hypoxemia, absence of CA, and COPD than did the surviving group. The dead group had lower levels of RBC count, Lymphocyte, count Baso count, and FT3 than did the surviving group. After variable assignment, the proportion of moderate-to-severe hypoxemia in the dead group and surviving group was 94.74% and 58.27%, respectively, the proportion receiving CA was 21.05% and 68.05%, respectively, the proportion of RBC <3.8 × 10^12^/L was 21.05% and 3.38%, respectively, and the proportion of D-dimer ≥280 ng/mL was 68.42% and 16.54%, respectively. Comparison of original data between the two groups is presented in Table 4, and post-assignment comparisons can be found in Table 5.

**Table 4.** **Analysis of original data contrasting survival and death groups in the training set**.

Variables		Total (n = 285)	Survival (n = 266)	Death (n = 19)	*t*/Z/χ^2^	*p*
RBC, mean (SD)		4.74 (0.61)	4.78 (0.60)	4.26 (0.53)	3.63	<0.001
Lymphocyte, Med (Q_1_, Q_3_)		1.91 (1.48, 2.28)	1.93 (1.50, 2.31)	1.41 (1.10, 1.79)	–3.38	<0.001
Baso, Med (Q_1_, Q_3_)		0.03 (0.02, 0.05)	0.03 (0.02, 0.05)	0.02 (0.01, 0.04)	–2.89	0.004
TBA, Med (Q_1_, Q_3_)		3.89 (2.29, 6.42)	3.88 (2.32, 6.29)	5.00 (2.12, 7.80)	–0.81	0.418
D-dimer, Med (Q_1_, Q_3_)		84.00 (46.00, 181.00)	78.00 (44.00, 152.50)	371.00 (185.00, 711.50)	–4.75	<0.001
FT3, Med (Q_1_, Q_3_)		4.57 (4.10, 5.11)	4.57 (4.13, 5.12)	3.53 (2.65, 4.31)	–4.16	<0.001
NT-proBNP, Med (Q_1_, Q_3_)		737.00 (182.00, 1960.00)	646.80	1570.00	–2.85	0.004
			(169.35, 1883.50)	(748.50, 2735.00)		
Death, *n* (%)					269.16	<0.001
	No	266 (93.33)	266 (100.00)	0 (0.00)		
	Yes	19 (6.67)	0 (0.00)	19 (100.00)		
Hypoxemia, *n* (%)					9.89	0.002
	Mild	112 (39.30)	111 (41.73)	1 (5.26)		
	Moderate & Severe	173 (60.70)	155 (58.27)	18 (94.74)		
CA, *n* (%)					17.19	<0.001
	No	100 (35.09)	85 (31.95)	15 (78.95)		
	Yes	185 (64.91)	181 (68.05)	4 (21.05)		
COPD, *n* (%)					16.24	<0.001
	No	266 (93.33)	253 (95.11)	13 (68.42)		
	Yes	19 (6.67)	13 (4.89)	6 (31.58)		

Notes: *t*, *t*-test; Z, Mann-Whitney test; χ^2^, Chi-square test; SD, standard deviation; Med, median; Q_1_, 1st Quartile; Q_3_, 3st Quartile; CA, catheter ablation; RBC, red blood cell; Baso, basophil granulocyte; TBA, total bile acids; FT3, free triiodothyronine; COPD, chronic obstructive pulmonary disease; NT-proBNP, N-terminal pro-brain natriuretic peptide.

**Tbale 5.** **Comparison of dummy variable assignment in the training set between survival and death groups**.

Variables		Total (n = 285)	Survival (n = 266)	Death (n = 19)	χ^2^	*p*
CA, *n* (%)					17.19	<0.001
	No	100 (35.09)	85 (31.95)	15 (78.95)		
	Yes	185 (64.91)	181 (68.05)	4 (21.05)		
Hypoxemia, *n* (%)					9.89	0.002
	Mild	112 (39.30)	111 (41.73)	1 (5.26)		
	Moderate & Severe	173 (60.70)	155 (58.27)	18 (94.74)		
RBC, *n* (%)					14.44	<0.001
	[3.8, 5.1]	191 (67.02)	178 (66.92)	13 (68.42)		
	<3.8	13 (4.56)	9 (3.38)	4 (21.05)		
	>5.1	81 (28.42)	79 (29.70)	2 (10.53)		
Lymphocyte, *n* (%)					-	0.023
	[1.1, 3.2]	251 (88.07)	237 (89.10)	14 (73.68)		
	<1.1	23 (8.07)	18 (6.77)	5 (26.32)		
	>3.2	11 (3.86)	11 (4.14)	0 (0.00)		
Baso, *n* (%)					1.03	0.309
	≤0.06	259 (90.88)	240 (90.23)	19 (100.00)		
	>0.06	26 (9.12)	26 (9.77)	0 (0.00)		
TBA, *n* (%)					0.4	0.527
	≤10	259 (90.88)	243 (91.35)	16 (84.21)		
	>10	26 (9.12)	23 (8.65)	3 (15.79)		
D-dimer, *n* (%)					26.68	<0.001
	<280	228 (80.00)	222 (83.46)	6 (31.58)		
	≥280	57 (20.00)	44 (16.54)	13 (68.42)		
FT3, *n* (%)					-	<0.001
	[3.1, 6.8]	268 (94.04)	255 (95.86)	13 (68.42)		
	<3.1	16 (5.61)	10 (3.76)	6 (31.58)		
	>6.8	1 (0.35)	1 (0.38)	0 (0.00)		
NT-proBNP, *n* (%)					1.54	0.215
	≤125	53 (18.60)	52 (19.55)	1 (5.26)		
	>125	232 (81.40)	214 (80.45)	18 (94.74)		
COPD, *n* (%)					16.24	<0.001
	No	266 (93.33)	253 (95.11)	13 (68.42)		
	Yes	19 (6.67)	13 (4.89)	6 (31.58)		

Notes: χ^2^, Chi-square test; -, Fisher exact; CA, catheter ablation; RBC, red blood cell; Baso, basophil granulocyte; TBA, total bile acids; FT3, free triiodothyronine; COPD, chronic obstructive pulmonary disease; NT-proBNP, N-terminal pro-brain natriuretic peptide.

### 3.4 Results of Single-Factor and Multiple-Factor Logistic Regression in the Training Set

Univariate logistic regression analysis revealed that CA (odds ratio (OR) = 0.13) was a potential protective factor, whereas moderate-to-severe hypoxemia (OR = 12.89), RBC count <3.8 × 10^12^/L (OR = 6.09), Lymphocyte count <1.1 × 10^9^/L (OR = 4.7), D-dimer ≥280 ng/mL (OR = 10.93), FT3 <3.1 pmol/L (OR = 11.77), and COPD (OR = 8.98) were potential risk factors. Multivariate logistic regression analysis showed that CA (OR = 0.21) was an independent protective factor, whereas moderate-to-severe hypoxemia (OR = 11.11), RBC count <3.8 × 10^12^/L (OR = 20.70), and D-dimer ≥280 ng/mL (OR = 7.07) were independent risk factors. The results of univariate and multivariate logistic regression analyses are presented in Table 6.

**Table 6.** **Presentation of single-factor and multiple-factor logistic regression results in the training set**.

Variables		Single-factor					Multiple-factor				
		β	S.E	Z	*p*	OR (95% CI)	β	S.E	Z	*p*	OR (95% CI)
CA											
	No					1.00 (Reference)					1.00 (Reference)
	Yes	–2.08	0.58	–3.6	<0.001	0.13 (0.04~0.39)	–1.57	0.69	–2.26	0.024	0.21 (0.05~0.81)
Hypoxemia											
	Mild					1.00 (Reference)					1.00 (Reference)
	Moderate & Severe	2.56	1.03	2.47	0.013	12.89 (1.70~97.98)	2.41	1.09	2.21	0.027	11.11 (1.32~93.62)
RBC											
	[3.8, 5.1]					1.00 (Reference)					1.00 (Reference)
	<3.8	1.81	0.67	2.71	0.007	6.09 (1.65~22.45)	3.03	0.93	3.25	0.001	20.70 (3.33~128.53)
	>5.1	–1.06	0.77	–1.37	0.17	0.35 (0.08~1.57)	–0.9	0.83	–1.09	0.274	0.41 (0.08~2.04)
Lymphocyte											
	[1.1, 3.2]					1.00 (Reference)					
	<1.1	1.55	0.58	2.69	0.007	4.70 (1.52~14.53)					
	>3.2	–14.74	1192.83	–0.01	0.99	0.00 (0.00~Inf)					
Baso											
	≤0.06					1.00 (Reference)					
	>0.06	–16.03	1279.19	–0.01	0.99	0.00 (0.00~Inf)					
D-dimer											
	<280					1.00 (Reference)					1.00 (Reference)
	≥280	2.39	0.52	4.6	<0.001	10.93 (3.94~30.32)	1.96	0.64	3.06	0.002	7.07 (2.02~24.74)
TBA											
	≤10					1.00 (Reference)					
	>10	0.68	0.67	1.03	0.305	1.98 (0.54~7.31)					
FT3											
	[3.1, 6.8]					1.00 (Reference)					
	<3.1	2.47	0.59	4.18	<0.001	11.77 (3.71~37.37)					
	>6.8	–12.59	1455.4	–0.01	0.993	0.00 (0.00~Inf)					
NT-proBNP											
	≤125					1.00 (Reference)					
	>125	1.48	1.04	1.42	0.155	4.37 (0.57~33.51)					
COPD											
	No					1.00 (Reference)					1.00 (Reference)
	Yes	2.2	0.57	3.85	<0.001	8.98 (2.94~27.43)	1.19	0.68	1.76	0.078	3.29 (0.87~12.42)

Notes: OR, odds ratio; CI, confidence interval; CA, catheter ablation; RBC, red blood cell; Baso, basophil granulocyte; TBA, total bile acids; FT3, free triiodothyronine; COPD, chronic obstructive pulmonary disease; NT-proBNP, N-terminal pro-brain natriuretic peptide.

### 3.5 Evaluation and Presentation of the Predictive Model for All-Cause Mortality of Patients With AF + OSAS

**Fig. 3.**
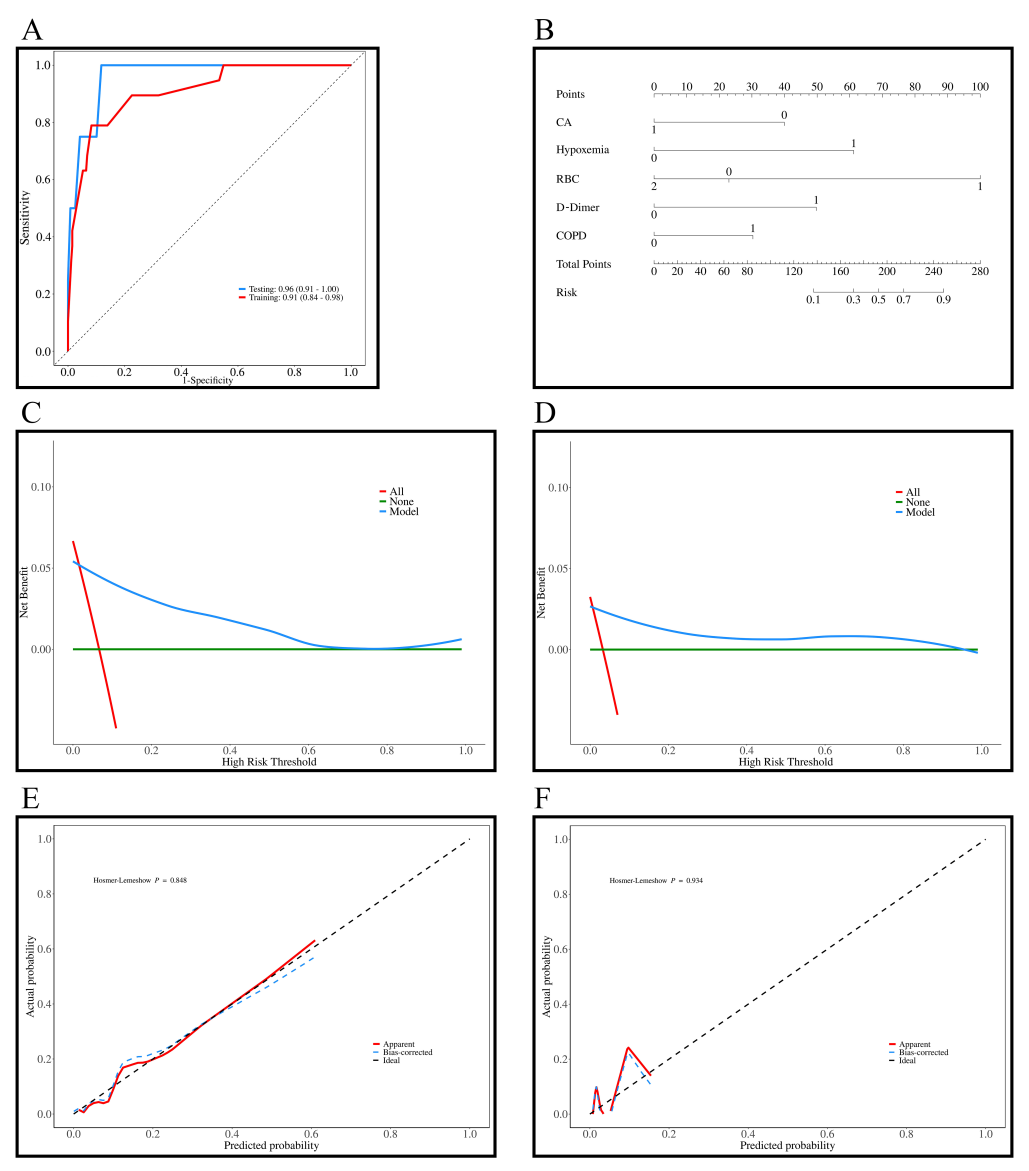
**Results of the prediction model for all-cause mortality in patients with AF + OSAS**. (A) ROC curves of the training (*n* = 285) and testing sets (*n* = 123); (B) Diagnostic nomogram of the all-cause mortality model; (C) DCA curves of the training set; (D) DCA curves of the testing set; (E) Diagnostic calibration curves of the training set; (F) Diagnostic calibration curves of the testing set. AF, atrial fibrillation; DCA, decision curve analysis; ROC, receiver operating characteristic; OSAS, obstructive sleep apnea syndrome; CA, catheter ablation; RBC, red blood cell; COPD, chronic obstructive pulmonary disease.

The AUC values for the training and testing sets were 0.91 (95% CI: 0.84–0.98) and 0.96 (95% CI: 0.91–1.00), indicating excellent predictive ability of the model (Fig. 3A). In Fig. 3B, the nomogram was developed based on the multivariable logistic regression, with each line corresponding to CA, hypoxemia, RBC count, D-dimer, and COPD, among which RBC count showed the most significant impact on the predicted outcomes. Fig. 3C,D show the DCA curves for the training and testing sets, and show that that the predictive model performs well within probability thresholds of 0.1 to 0.7 and 0.1 to 0.9, respectively. Fig. 3E,F show the diagnostic calibration curves for the training and validation sets, which further confirm the stability and accurate predictive performance of the model.

The assessment results of the model for all-cause mortality in patients with AF + OSAS in the training and testing sets are as follows: The model was tested using a likelihood ratio test, with both the training and testing sets showing significance, with *p*-values of 2.2 × 10^-8^ and 0.0230, respectively, indicating overall model significance. The C-indexes for the training and testing sets were 0.912 (0.844–0.980) and 0.716 (0.46–0.967), respectively, indicating high accuracy of the model. The goodness-of-fit tests for calibration evaluation showed *p*-values of 0.8054 and 1.000 for the training and testing sets, respectively; both were greater than 0.05, indicating good model fit. Detailed data can be found in Table 7. In the present study, the training-set and testing-set Brier Scores were 0.044 and 0.056, respectively, indicating a high level of accuracy in model predictions. The calibration slopes were 1.068 and 1.341, respectively, suggesting a high level of consistency between predicted probabilities and actual event rates. Specific results are detailed in Table 8.

**Table 7.** **Model evaluation of patients with AF + OSAS in the training and testing sets**.

Evaluation direction	Evaluation content	Train		Test	
		Statistics	*p*	Statistics	*p*
Model verification	Likelihood ratio test	χ^2^: 55.838	2.2 × 10^–⁢8^	χ^2^: 5.1662	0.0230
Discrimination assessment	C-index	C-index: 0.912 (0.844–0.980)	1.82 × 10^–⁢33^	C-index: 0.716 (0.465–0.967)	0.0847
Calibration assessment	Goodness-of-fit test	χ^2^: 4.54	0.8054	χ^2^: 1.6921 × 10^–⁢15^	1.000

Notes: χ^2^, Chi-square test; AF, atrial fibrillation; OSAS, Obstructive sleep apnea syndrome.

**Table 8.** **Calibration-performance results of the training and testing sets**.

	Train	Test
Brier score	0.044	0.056
Calibration slope	1.068	1.341

The AUC for the training and testing sets were 0.91 and 0.96, with accuracies of 0.91 and 0.91. The sensitivity and specificity of the training set were 0.92 and 0.79, and for the testing set were 0.92 and 0.75. The positive predictive value (PPV) for the training and testing sets were 0.98 and 0.99, and the negative predictive value (NPV) were 0.41 and 0.23, respectively. These results indicated that the model exhibited good predictive performance for all-cause mortality in patients with AF + OSAS. The confusion-matrix results for the training and testing sets can be found in Table 9.

**Table 9.** **Confusion-matrix results of the training and testing sets**.

Data	AUC (95% CI)	Accuracy (95% CI)	Sensitivity (95% CI)	Specificity (95% CI)	PPV (95% CI)	NPV (95% CI)	Cut off
Train	0.91 (0.84–0.98)	0.91 (0.87–0.94)	0.92 (0.88–0.95)	0.79 (0.61–0.97)	0.98 (0.97–1.00)	0.41 (0.25–0.56)	0.142
Test	0.96 (0.91–1.00)	0.91 (0.85–0.95)	0.92 (0.87–0.97)	0.75 (0.33–1.00)	0.99 (0.97–1.00)	0.23 (0.00–0.46)	0.142

Notes: AUC, area under the curve; PPV, positive predictive value; NPV, negative predictive value; CI, confidence interval.

### 3.6 Scoring Table and Risk Assessment for All-Cause Mortality of Patients With AF + OSAS

**Fig. 4.**
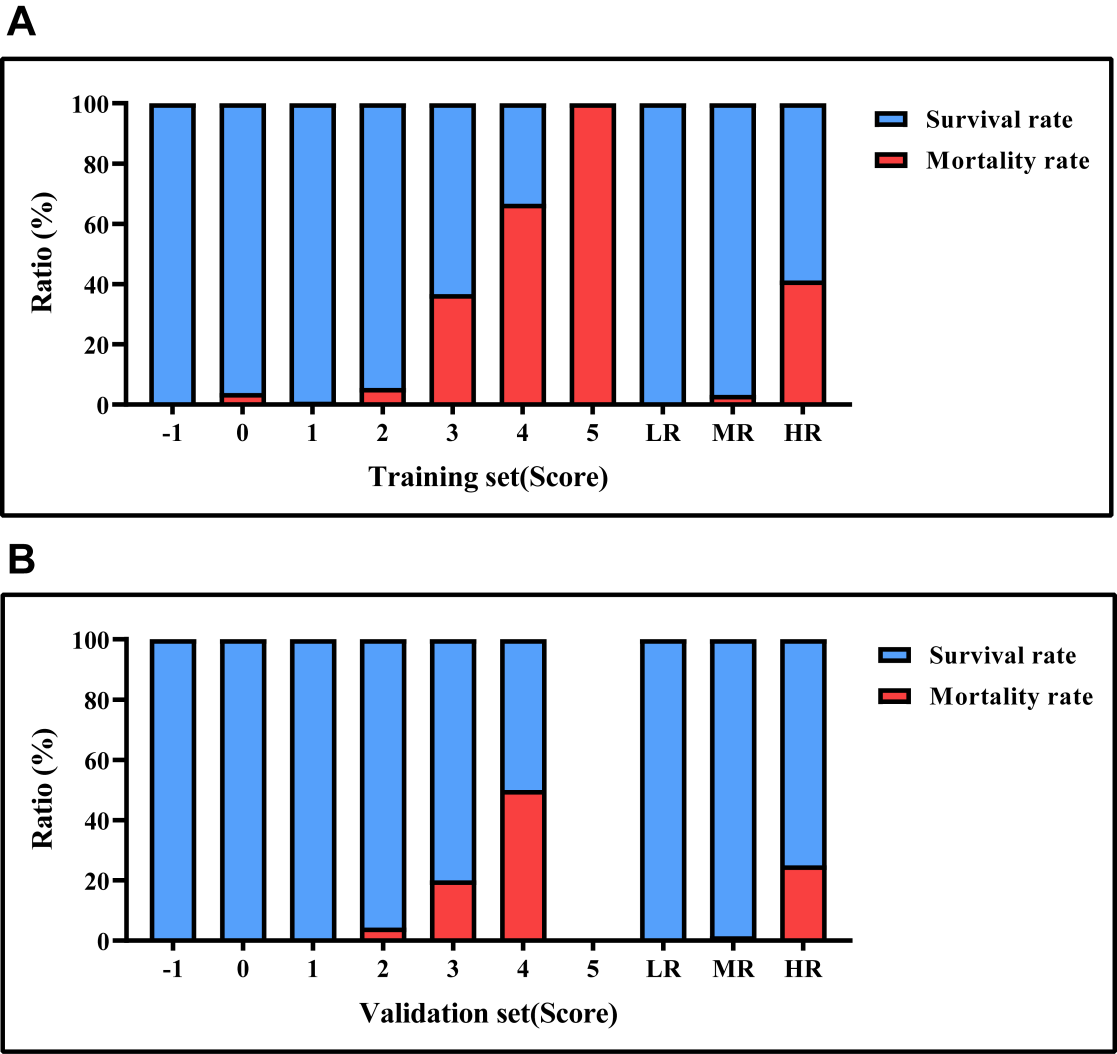
**Mortality rates based on risk score values**. (A) the survival and mortality rates for each risk level in the training set. (B) the survival and mortality rates for each risk level in the testing set. LR, low risk; MR, medium risk; HR, high risk.

An all-cause mortality-risk scoring system for patients with AF + OSAS was developed based on the Framingham method, where CA was assigned –1 point, moderate-to-severe hypoxemia was assigned 2 points, RBC <3.8 × 10^12^/L was assigned 2 points, and D-dimer ≥280 ng/mL was assigned 1 point. The assignment of each independent risk factor is provided in Table 10. The training and testing sets were evaluated based on this scoring system. In this study, scores ranging from –1 to 0 points were classified as low risk, 1 to 2 points as moderate risk, and 3 to 6 points as high risk. In the percentage bar chart, note that as the score increases, the mortality rate of patients increases and the survival rate decreases. The scoring results for the training and testing sets can be found in Table 11, and the bar chart comparing all-cause mortality rates by score is presented in Fig. 4.

**Table 10.** **Risk scoring table for all-cause mortality of patients with AF + OSAS**.

Characteristics	Categories	Points
CA	Yes	–1
Hypoxemia	Moderate & Severe	2
RBC	<3.8 × 10^12^/L	2
D-dimer	≥280 ng/mL	1

Notes: CA, catheter ablation; RBC, red blood cell; AF, atrial fibrillation; OSAS, obstructive sleep apnea syndrome.

**Table 11.** **Death and survival rate of patients with AF + OSAS in the train and test sets at different scores**.

Points	Train				Test			
	Survival	Survival rate	Death	Death rate	Survival	Survival rate	Death	Death rate
–1	71	100.00%	0	0.00%	31	100.00%	0	0.00%
0	25	96.15%	1	3.85%	12	100.00%	0	0.00%
1	99	99.00%	1	1.00%	45	100.00%	0	0.00%
2	51	94.44%	3	5.56%	22	95.65%	1	4.35%
3	19	63.33%	11	36.67%	8	80.00%	2	20.00%
4	1	33.33%	2	66.67%	1	50.00%	1	50.00%
5	0	0.00%	1	100.00%	-	-	-	-
–1~0 points (low risk)	96	100.00%	0	0.00%	43	100.00%	0	0.00%
1~2 points (medium risk)	150	96.77%	5	3.23%	67	98.53%	1	1.47%
3~5 points (high risk)	20	58.82%	14	41.18%	9	75.00%	3	25.00%

Notes: AF, atrial fibrillation; OSAS, Obstructive sleep apnea syndrome.

## 4. Discussion

The condition of AF + OSAS, due to its complexity and multitude of factors, has resulted in high mortality rates. Given the disease burden, families also have faced significant pressures, making it a substantial public health challenge. In the present study, through Lasso regression, we selected 10 variables from the abundant patient information that included hypoxemia, CA, RBC count, Lymphocyte count, Baso count, TBA, D-dimer, FT3, NT-proBNP, and COPD. Significant variables identified in the univariate logistic regression analysis were included in the multivariable logistic regression analysis, which revealed that CA (OR = 0.21) was an independent protective factor, whereas moderate-to-severe hypoxemia (OR = 11.11), RBC <3.8 × 10^12^/L (OR = 20.70), and D-dimer ≥280 ng/mL (OR = 7.07) were independent risk factors. A risk-scoring system was developed in the present study to assess the overall mortality risk of patients with AF + OSAS, which defined –1–0 points as low risk, 1–2 points as moderate risk, and 3–5 points as high risk. The percentage bar chart showed that as the risk level increased, the mortality rate of patients rose, and the survival rate decreased. The results of this study demonstrated good predictive accuracy.

Long-term irregular and rapid contractions of the left atrium easily lead to stroke and HF, which increases mortality. CA is the main effective treatment for AF. The present study found that CA is an independent protective factor for all-cause mortality in patients with AF + OSAS. CA can increase the duration of sinus rhythm maintenance by interrupting abnormal electrical impulses conduction and sustaining pulmonary vein isolation, thereby reducing the burden of AF and slowing the progression from paroxysmal AF to persistent AF, and decreasing cardiac workload. This significantly reduces the risk of mortality, HF, stroke, and rehospitalization in patients with AF [18, 19, 20]. Then, CA can actively improve patient anxiety levels and symptom burden, thereby enhancing the quality of life [21, 22]. Moreover, we speculate that in patients with AF + OSAS, the atrial arrhythmia may lead to impaired atrial contraction function and compromised blood circulation, further exacerbating the patient’s hypoxemia. Restoration of normal cardiac rhythm after CA ensures adequate oxygen supply by normalizing cardiac function and blood circulation, thereby improving oxygenation status and reducing all-cause mortality. Therefore, we recommend that patients with AF + OSAS undergo CA treatment promptly to restore and maintain sinus rhythm, thereby reducing the risk of related complications, and improving quality of life and prognosis.

Of note is the fact that multivariable logistic regression analysis identified moderate to severe hypoxemia and RBC <3.8 × 10^12^/L as independent risk factors for all-cause mortality in patients with AF + OSAS. In the first place, OSAS primarily manifests as recurrent episodes of sleep apnea, leading to periodic nocturnal hypoxemia and hypercapnia. RBCs, as the major carrier of oxygen in the blood, decrease because untreated OSAS-induced inflammatory stimulation of macrophages, which in turn reduces RBC levels [23] and exacerbates hypoxemia. This subsequently triggers the alternate activation of the sympathetic and vagal nervous systems, inducing inflammation, cardiac structural remodeling, interstitial fibrosis, and ion channel disturbances, resulting in prolonged action potential duration and decreased conduction velocity. These changes lead to the formation of focal activity and reentry, promoting the progression of AF [24, 25, 26]. Second, the deoxygenation caused by hypoxemia disrupts the balance of electron transfer in the electron transport chain, leading to sustained oxidative stress [27]. The intensified oxidative stress exacerbates mitochondrial dysfunction and cellular injury, triggering the activation of transcription factors and subsequently increasing the production of inflammatory mediators [28]. Third, hypoxemia can prolong the atrial refractory period, slow conduction, and increase conduction inhomogeneity, leading to atrial electrical remodeling and worsening AF. What is more, moderate to severe nocturnal hypoxemia during sleep can lead to heightened sympathetic nervous system activity, triggering vasoconstriction, elevated blood pressure, and increased heart rate [29, 30, 31], thereby increasing the patient’s demand for oxygen. However, the reduction in RBC levels caused by untreated OSAS exacerbates hypoxemia, which can lead to prolonged myocardial oxygen deficiency and potentially induce HF. Studies have shown that OSAS is independently associated with an increased all-cause mortality rate [32], and severe nocturnal hypoxemia can independently predict sudden cardiac death [33]. CPAP, as the primary treatment for OSAS, can alleviate the burden of AF after CA or cardiac resynchronization and slow down the progression of AF [34, 35]. Despite methodological limitations and small sample sizes, these studies largely supported the view that CPAP can alleviate the burden of AF and may thereby improve prognosis. Furthermore, we speculate that severe nocturnal hypoxemia may lead to systemic hypoxia, increasing the risk of other cardiovascular events, thus further worsening prognosis. Given that many patients with AF + OSAS do not exhibit daytime symptoms of excessive sleepiness, the presence of OSAS may be masked, resulting in underdiagnosis and undertreatment of OSAS. Therefore, we recommend that symptomatic AF patients undergo proactive screening for OSAS to facilitate early detection, diagnosis, and treatment with CA and CPAP, thereby enhancing long-term prognosis.

In clinical practice, elevated levels of the fibrin-degradation product D-dimer are commonly used to assess the risk of thromboembolism and serve as an independent risk factor for all-cause mortality in patients with AF + OSAS. Primarily, the disordered intra-atrial electrical activity in patients with AF results in ineffective atrial contractions, leading to blood stasis in the atria and facilitating the formation of atrial appendage thrombi. If there are disturbances in the contractile and relaxation functions of the left ventricle, this further exacerbates left atrial enlargement and congestion, thereby promoting thrombus formation. Once these thrombi dislodge, they may trigger thromboembolic events, potentially causing severe conditions such as stroke [36]. Secondarily, the coexistence of AF and OSAS can trigger nocturnal hypoxemia, which not only increases blood viscosity, leading to circulatory disturbances, but also promotes thrombus formation, thereby adversely affecting patient prognosis. Moreover, AF + OSAS can cause dysfunction of endothelial cells, disrupting the stability of the vessel wall, increasing the risk of intravascular thrombosis, and consequently raising the likelihood of developing cardiovascular and cerebrovascular diseases [37]. Previous studies have shown that plasma D-dimer levels are significantly higher in patients with AF [38], and are significantly associated with stroke and cardiovascular death in AF patients [38, 39]. OSAS is significantly associated with thrombus formation [40, 41]. Rivaroxaban can effectively reduce plasma D-dimer levels in patients [42]. Therefore, we recommend that when AF + OSAS patients exhibit elevated D-dimer levels, consideration should be given to identifying the location of the thrombus and initiating or intensifying anticoagulant therapy. Additionally, timely correction of reversible risk factors can help reduce the risk of thromboembolism in these patients. Elevation of D-dimer, a marker of coagulation-fibrinolysis system activation, may reflect the hypercoagulable state and hidden thrombotic burden in patients with AF + OSAS. It is noteworthy that anticoagulant therapy may partially mask the predictive value of D-dimer, and systemic inflammation may interact bidirectionally with D-dimer formation through procoagulant effects. Despite the adjustments made to reduce confounding bias through multivariable analysis in the present study, future prospective research combining dynamic D-dimer monitoring and analysis of inflammatory markers is essential to clarify its independent predictive role.

The scoring system developed in the present study suggested that in clinical practice, for patients with AF, enhanced screening for OSAS using polysomnography (PSG) should be conducted. If both conditions exist, routine blood counts and D-dimer tests should be performed. Additionally, the development of embedded plugins in electronic medical records can automatically score relevant results and stratify patients based on their scores, while providing alerts to supervising physicians. Furthermore, tailored management strategies should be implemented based on patients’ different risk stratifications: for low-risk patients, outpatient management with remote treatment compliance monitoring every six months; for moderate-risk patients, multidisciplinary team assessments should be initiated—for example, those patients may require multidisciplinary team (MDT) evaluations to identify and address potential underlying causes of OSAS, along with cardiology consultations for CA surgery. High-risk patients should have shorter follow-up intervals and increased outpatient-visit frequency through communication channels like phone calls, texts, and video conferencing. Equipping patients’ homes with devices such as pulse oximeters and smartwatches to monitor basic vital signs, and enhancing education for patients’ families on disease awareness and fundamental management strategies, is advised.

The conclusions drawn from this retrospective study are multidimensional. First, the study identified key factors influencing all-cause mortality in patients with AF + OSAS, encompassing both protective and risk factors. Second, the study developed a model for predicting the risk of all-cause mortality in patients with AF + OSAS, demonstrating good predictive performance. Finally, the scoring system developed based on this predictive model provided an effective tool for assessing and predicting AF recurrence, offering valuable guidance and reference for future clinical management.

## 5. Strengths and Limitations

Patients with AF + OSAS face a higher risk of mortality. The complex pathophysiological mechanisms of this condition have garnered significant attention. With the aging population, the incidence rates of OSAS + AF continue to rise, undoubtedly placing a heavy burden on the public health system. To our knowledge, there is currently a lack of specific tools for assessing the overall risk of mortality in patients with AF + OSAS. Therefore, this study developed a clinical predictive model suitable for this patient population. However, the study still has its limitations. First, although data from AF + OSAS patients who received treatment between 2012 and 2024 were collected retrospectively in this study, the sample size was relatively small, with a low number of deceased patients, leading to the Effective Predictor Variable being lower than the traditionally recommended value. This may affect the stability of the results. Despite rigorous variable selection, cross-validation, and independent validation, the model demonstrated good discriminative and calibration performance; however, this remains a limitation of the study. Second, although the hospital was the one with the highest number of patients in the region and thus considered representative, the predictive model was only internally validated in a single center. These limitations highlight the need for future research efforts to conduct multicenter prospective real-world clinical studies to validate these findings and provide assistance to a broader range of patients and healthcare professionals.

## 6. Conclusions

In patients with AF + OSAS, CA (OR = 0.21) is an independent protective factor for all-cause mortality, whereas moderate to severe hypoxemia (OR = 11.11), RBC <3.8 × 10^12^/L (OR = 20.70), and D-dimer ≥280 ng/mL (OR = 7.07) are independent risk factors. When these factors are combined in a scoring system, they demonstrate good predictive performance.

## Availability of Data and Materials

The datasets used and analyzed during the current study are available from the corresponding author on reasonable request.
